# Frailty Increases Morbidity and Mortality in Patients Undergoing Oncological Liver Resections: A Systematic Review and Meta-analysis

**DOI:** 10.1245/s10434-024-15571-8

**Published:** 2024-06-10

**Authors:** Sorinel Lunca, Stefan Morarasu, Kevin Rouet, Andreea Antonina Ivanov, Bianca Codrina Morarasu, Cristian Ene Roata, Cillian Clancy, Gabriel-Mihail Dimofte

**Affiliations:** 1 2nd Department of Surgical Oncology, Regional Institute of Oncology (IRO), Iasi, Romania; 2 Grigore T Popa University of Medicine and Pharmacy Iasi, Iasi, Romania; 3 Department of Internal Medicine and Toxicology, Saint Spiridon University Regional Emergency Hospital, Iasi, Romania; 4 Department of Colorectal Surgery, Tallaght University Hospital, Dublin 24, Ireland

**Keywords:** Frailty, Hepatectomy, Liver cancer, Surgical oncology, Morbidity

## Abstract

**Background:**

Considered to reflect a patients’ biological age, frailty is a new syndrome shown to predict surgical outcomes in elderly patients. In view of the increasing age at which patients are proposed oncological liver surgery and the morbidity associated with it, we attempted to perform a systematic review and meta-analysis to compare morbidity and mortality between frail and nonfrail patients after liver resections.

**Methods:**

The study was registered with PROSPERO. A systematic search of PubMed and EMBASE databases was performed for all comparative studies examining surgical outcomes after liver resections between frail and nonfrail patients.

**Results:**

Ten studies were included based on the selection criteria with a total of 71,102 patients, split into two groups: frail (*n* = 17,167) and the control group (*n* = 53,928). There were more elderly patients with a lower preoperative albumin level in the frail group (*p* = 0.02, *p* = 0.001). Frail patients showed higher rates of morbidity with more major complications and a higher incidence of postoperative liver failure (*p* < 0.001). Mortality (*p* < 0.001) and readmission rate (*p* = 0.021) also was higher in frail patients.

**Conclusions:**

Frailty seems to be a solid predictive risk factor of morbidity and mortality after liver surgery and should be considered a selection criterion for liver surgery in at-risk patients.

Life expectancy around the world has increased steadily and constantly. This will continue as the mean age is expected to increase to 77.3 years by 2050.^1,2^ This exerts pressure on the surgical services, because more and more elderly patients will require surgery and their postoperative outcomes must be weighted with more caution. We must consider that roughly one in seven elderly patients die in the first year after major surgery.^3^

When considering oncological liver surgery in such patients, which is one of the most complex and high-risk abdominal interventions, we must be even more pragmatic. Considering elderly patients for major liver resection is difficult, because age is not a clear-cut risk factor, but rather one must consider the patients` general status and response to surgical stress, which can be objectively quantified through frailty. Frailty is a structured syndrome characterized by a decreased potential to handle physiological stress.^4^ It is a clinical entity capable of selecting patients who may have more difficulties in maintaining homeostasis after stress, including surgical trauma.^4–6^ While frailty is driven by aging, it is not always present in elderly patients.

If deemed operable, in primary liver malignancies and colorectal liver metastases, surgery is the mainstay of treatment; therefore, even frail patients are considered candidates. To enable better stratification and help decision making, several clinical judgement-based scores have been validated to quantity frailty by analysing the patients’ general status, cognition, and comorbidities. A higher frailty score should predict worse outcomes and thus help both surgeons and patients to decide their treatment and understand expectations. Our goal was to gather all comparative data, analyze the role of frailty in predicting outcomes after major liver resections, and compare them in a meta-analytical model, thus consolidating the role of preoperative frailty workup for liver surgery candidates.

## Materials and Methods

### Literature Search and Study Selection

**Fig. 1:**
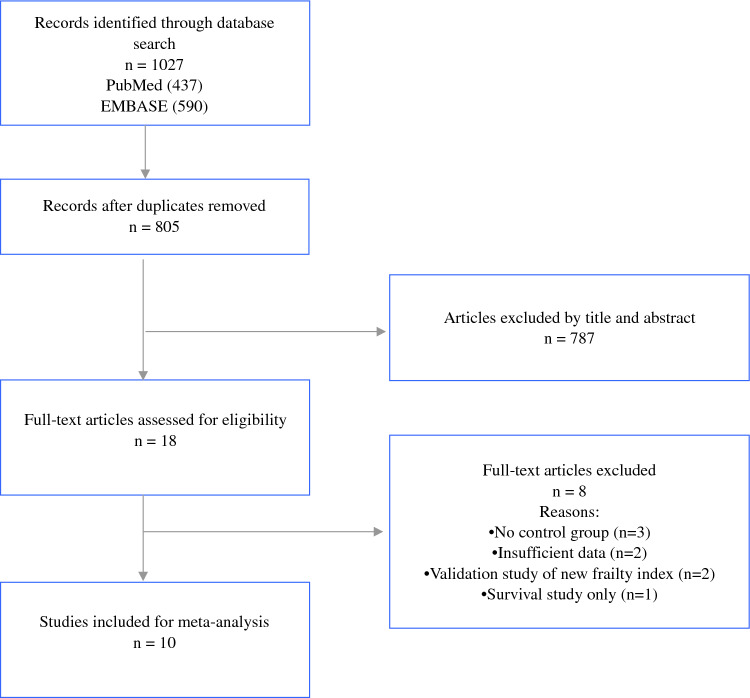
PRISMA diagram. Preferred reporting items in systematic reviews and meta-analysis

**Fig. 2:**
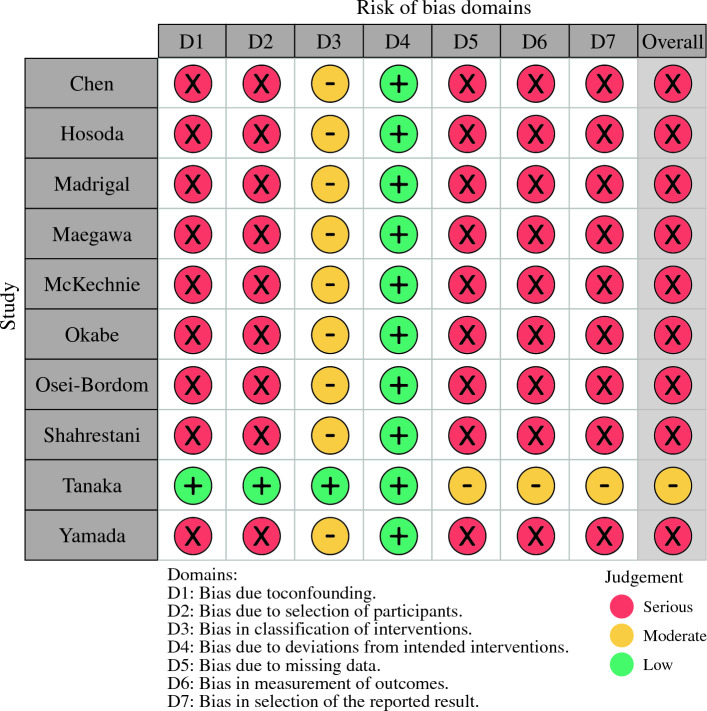
ROBINS-I risk of bias assessment. Assessment of risk of bias was done by two authors (SM and CC). RCTs were analysed via the ROBINS I tool. Each study was classified as low/moderate/serious risk for each of the five or seven domains. Disagreements were resolved via consensus

The study was registered with PROSPERO (International Prospective Register of Systematic Reviews). The study ID is CRD42024510933. A systematic search of PubMed and EMBASE databases was performed for all comparative studies examining surgical outcomes in patients who underwent oncological liver resection and had their frailty index measured preoperatively. The following search algorithm was used: (frail) AND (liver OR hepatic) AND (surgery OR resection OR operation). Preferred Reporting Items for Systematic Reviews and Meta-Analyses (PRISMA) guidelines were used as search protocol and the PRISMA checklist was followed to conduct the methodology (Fig. 1).^7^ Inclusion criteria were used according to the Problem, Intervention, Comparison, and Outcome (PICO) formula. The latest search was performed on January 5, 2024. Two authors (SM and SL) assessed the titles and abstracts of studies found in the search, and the full texts of potentially eligible trials were reviewed. Disagreements were resolved by consensus-based discussion. The Newcastle-Ottawa scale (Table 1) and the ROB2 and ROBINS-I tools (Fig. 2) were used to quantify quality of eligible studies as previously done.^8–11^ The references of full texts reviewed were further screened for additional eligible studies. The corresponding author was contacted to clarify data extraction if additional information was necessary.

**Table 1:** Overview of included studies

Author	Year of publication	Type of study	Frailty score used	When was frailty scored?	Total no. patients	F no	NF no	NOS
Chen [13]	2017	Retrospective	5-mFI	Retrospectively	1928	225	1706	7
Hosoda [14]	2022	Retrospective	CFS	Retrospectively	87	44	35	7
Madrigal [15]	2022	Retrospective	JHS	Retrospectively	40735	3655	37080	7
Maegawa [16]	2022	Retrospective	11-mFI	Retrospectively	24150	11687	12463	7
McKechnie [17]	2020	Retrospective	11-mFI	Retrospectively	409	58	351	7
Okabe [18]	2019	Retrospective	CFS	Prospectively	143	16	127	7
Osei-Bordom [19]	2022	Retrospective	11-mFI	Prospectively	1826	632	1192	7
Shahrestani [20]	2023	Retrospective	JHS	Prospectively	1515	766	749	7
Tanaka [21]	2018	Prospective	KCL	Prospectively	217	63	154	7
Yamada [22]	2021	Prospective	CFS	Prospectively	92	21	71	7

*F* frail; *NF* nonfrail; *NOS* Newcastle-Ottawa Scale; *5-mFI* 5-item modified frailty index; *CFS* clinical frailty scale; *JHS* Johns Hopkins score; *11-mFI* 11-item modified frailty index; *KCL* Kihon checklist

### Eligibility Criteria

Studies written in English, including comparative surgical data between frail versus nonfrail patients, were assessed for eligibility. The primary endpoints were general morbidity, rate of major complications (Clavien Dindo more than IIIa), mortality, and length of stay. Studies without comparative data were not included. Studies that included data only on survival, rather than morbidity, were excluded.

### Data Extraction and Outcomes

For each eligible study the following data were recorded: author’s names, journal, year of publication, study type, total number of patients and number of patients included in each group, frailty score used, age, body mass index (BMI), ASA score, preoperative comorbidities (respiratory, metabolic, cardiovascular), preoperative albumin and hemoglobin level, Child Pugh score, smoking status, preoperative chemotherapy, use of steroids, overall morbidity, surgical complications and type, medical complications and type, length of stay, length of intensive care unit (ICU) stay, mortality, and readmissions. For each study, the outcomes of interest were extracted and grouped into three main categories, which were further analysed: (1) Patients characteristics (age, BMI, preoperative albumin level); (2) Morbidity (general morbidity, major complications, Clavien-Dindo more than IIIa, surgical complications, postoperative liver failure); and (3) 30-day outcomes (length of stay, readmissions, mortality).

### Statistical Analysis

Random-effects models were used to measure all pooled outcomes as described by Der Simonian and Laird,^12^ and the odds ratio (OR) was estimated with its variance and 95% confidence interval (CI). The random effects analysis weighed the natural logarithm of each study's OR by the inverse of its variance plus an estimate of the between-study variance in the presence of between-study heterogeneity. Heterogeneity between ORs for the same outcome between different studies was assessed using the I^2^ inconsistency test and chi-square-based Cochran’s Q statistic test in which *p* < 0.05 is taken to indicate the presence of significant heterogeneity. For the main outcomes, publication bias was addressed by using the trim and fill method. Computations were performed by using RevMan 5.3 and Comprehensive Meta-Analysis Version 4 (for publication bias and Egger’s regression intercept).

## Results

### Eligible Studies

Ten studies containing surgical data after liver surgery between frail versus nonfrail patients were included (Table 1).^13–22^ The initial search found 1027 studies. After excluding duplicates and unrelated studies based on abstract triage, 18 full texts were assessed for eligibility, of which ten matched the inclusion criteria and were systematically reviewed. The year of publication of included studies ranged from 2017 to 2022. Eight studies were retrospective, whereas two were designed prospectively.^13–22^ The total number of included patients was 71,102, split into two groups: study group/frail group (F, *n* = 17,167) and control group/nonfrail group (NF, *n* = 53,928). Frailty was defined by using one of the already known scores: the 11-factor modified frailty index (mFI-11, *n* = 3), the 5-factor modified frailty index (mFI-5, *n* = 1), the clinical frailty scale (CFS, *n* = 3), the Johns Hopkins frailty assessment calculator (Johns Hopkins score, *n* = 2), and the Kihon checklist (KCL, *n* = 1). The mean age in the F was 72.3 ± 6.85 versus 69.4 ± 7.73 in the NF. Mean BMI was 25 ± 3.97 in the F versus 24.4 ± 4.55 in the NF. The mean preoperative albumin level, as a marker of nutritional status, was 3.6 ± 0.56 in the F and 3.8 ± 0.58 in the NF.

### Patients’ Characteristics

#### Age

**Fig. 3:**
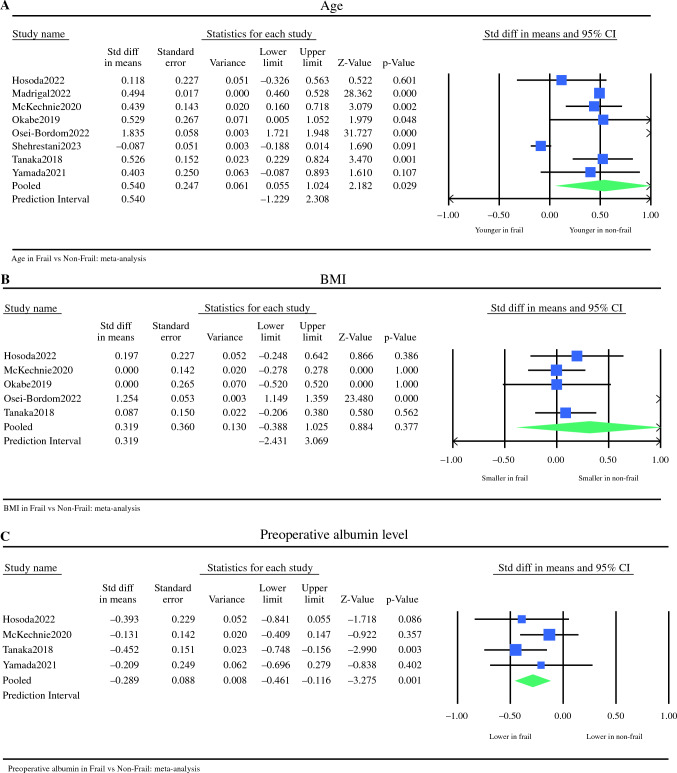
Meta analysis of patients’ characteristics: (**A**) Age; (**B**) BMI; (**C**) Preoperative albumin level. Each study is shown by the point estimate of the odds ratio/mean difference (OR; square proportional to the weight of each study) and 95% confidence interval (CI) for the OR (extending lines); the combined ORs/mean difference and 95% CIs by random effects calculations are shown by diamonds. (**A**) F versus NF and age (n = 45,014, *p* = 0.02; test for heterogeneity Q = 661,067, *p* < 0.001, *I*^2^ = 99%). (**B**) F versus NF and BMI (*n* = 2672, *p* = 0.37; test for heterogeneity Q = 134.332, *p* < 0.001, *I*^2^ = 97%). (**C**) F versus NF and albumin level (*n* = 797, *p* = 0.001; test for heterogeneity Q = 2.718, *I*^2^ = 0%)

Eight studies, describing 45,014 patients, included data on mean age.^14,15,17–22^ Nonfrail patients were significantly younger with a mean difference of 0.54 years, with significant interstudy variance (mean difference 0.540, 95% CI 0.055–1.024, *p* = 0.02, Q = 661.067, *p* < 0.001, I^2^ = 99%) (Fig. 3a).

#### Body Mass Imdex

Five studies, including data on 2672 patients, described the mean BMI between the two groups.^14,17–19,21^ There was no significant difference in terms of mean BMI, however, with significant interstudy heterogeneity (mean difference 0.319, 95% CI −0.388 to 1.025, *p* = 0.37, Q = 134.332, *p* < 0.001, I^2^ = 97%) (Fig. 3b).

#### Preoperative Albumin Level

Four studies, including 797 patients, reported on the mean preoperative albumin level, as a marker of patients’ nutritional status.^14,17,21,22^ Frail patients had a significantly lower albumin level compared with NF patients, with a mean difference of 0.28, without variance between included studies (mean difference 0.289, 95% CI 0.116–0.461, *p* = 0.001, Q = 2.718, *I*^2^ = 0%) (Fig. 3c).

### Morbidity

#### Overall Morbidity

**Fig. 4:**
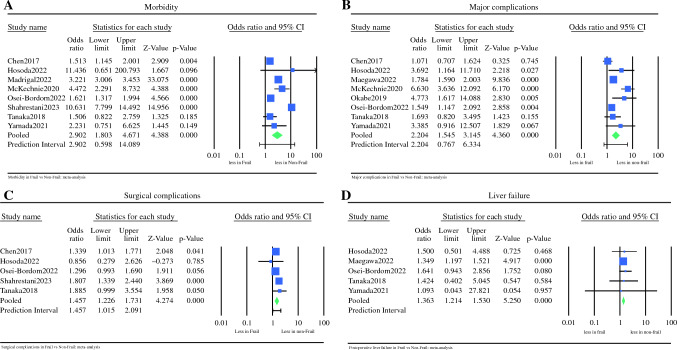
Meta analysis of morbidity between frail and non-frail patients in terms of (**A**) morbidity; (**B**) major complications; (**C**) surgical complications; (**D**) postoperative liver failure. Each study is shown by the point estimate of the odds ratio/mean difference (OR; square proportional to the weight of each study) and 95% confidence interval (CI) for the OR (extending lines); the combined ORs/mean difference and 95% CIs by random effects calculations are shown by diamonds. (**A**) F versus NF and morbidity (*n* = 46,806, *p* < 0.001; test for heterogeneity Q = 132.361, *p* < 0.001, *I*^2^ = 95%). (**B**) F versus NF and major complications (*n* = 28,849, *p* < 0.001; test for heterogeneity Q = 30.570, p < 0.001, *I*^2^ = 77%). (**C**) F versus NF and surgical complications (*n* = 5570, *p* < 0.001; test for heterogeneity Q = 4.580, *p* = 0.333, *I*^2^ = 13%). (**D**) F versus NF and postoperative liver failure (*n* = 26,366, *p* < 0.001; test for heterogeneity Q = 0.510, *I*^2^ = 0%)

**Fig. 5:**
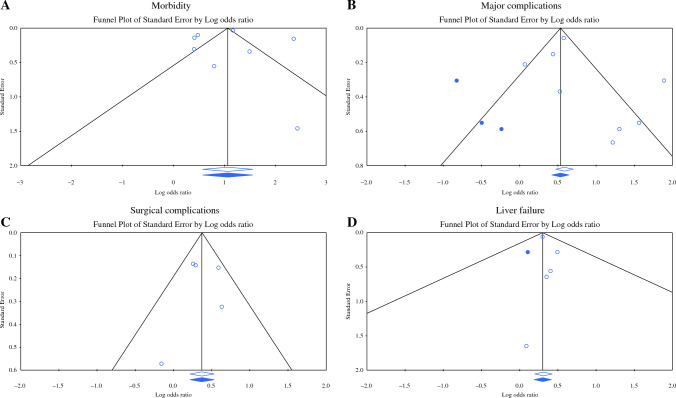
Trim and fill for morbidity: (**A**) morbidity; (**B**) major complications; (**C**) surgical complications; (**D**) postoperative liver failure. The funnel plot is a plot of a measure of study size (usually standard error or precision) on the vertical axis as a function of effect size on the horizontal axis. Large studies appear toward the top of the graph and tend to cluster near the mean effect size. Smaller studies appear toward the bottom of the graph. Through the trim and fill method studies were imputed to adjust for publication bias (full black circles). On the bottom of the graph, the empty diamond shows the OR and confidence interval for the original studies, while the full diamond shows the OR and confidence interval for the original and imputed studies

Eight studies,^13–15,17,19–22^ including data on 46,806 patients, compared overall morbidity between F and NF patients. Frail patients had a 2.9 times higher morbidity rate compared with NF patients (OR 2.902, 95% CI 1.803–4.671, *p* < 0.001, Q = 132.361, *p* < 0.001, *I*^2^ = 95%) (Fig. 4a). Under the random effects model, after trim and fill, there were no studies missing, and the overall results remaining unchanged (Fig. 5a).

#### Major Complications

Eight studies, including data on 28,849 patients, compared the rates of major complications (Clavien-Dindo more than IIIa) between the two groups.^13,14,16–19,21,22^ Frail patients had a 2.2 higher rate of major complications compared to NF patients (OR 2.204, 95% CI 1.545-3.145, *p* < 0.001, Q = 30.570, *p* < 0.001, *I*^2^ = 77%) (Fig. 4b). To reduce publication bias, three studies were imputed via the trim and fill method. Under the random effects model, the OR is 1.659 (95% CI 1.139–2.417) (Fig. 5b).

#### Surgical Complications

Five studies, including 5570 patients, extracted data relating to surgical complications between the two groups.^13,14,19–21^ Frail patients had a 1.4 times higher rate of surgical complications compared to NF patients, without significant interstudy heterogeneity (OR 1.457, 95% CI 1.226–1.731, *p* < 0.001, Q = 4.580, *p* = 0.333, *I*^2^ = 13%) (Fig. 4c). Under the random effects model, after trim and fill, there were no studies missing, and the overall results remaining unchanged (Fig. 5c).

### Postoperative Liver Failure

Five studies, including data on 26,366 patients, analysed the incidence of postoperative liver failure between F and NF patients.^14,16,19,21,22^ Frail patients had a 1.3 times higher rate of liver failure compared with NF, without significant interstudy variance (OR 1.363, 95% CI 1.214–1.530, *p* < 0.001, Q = 0.510, *I*^2^ = 0%) (Fig. 4d). Using the trim and fill method, one study was imputed to the left of the mean under the random effects model (OR 1.351, 95% CI 1.206–1.513) (Fig. 5d).

## Thirty-Day Outcomes

### Length of Stay

**Fig. 6:**
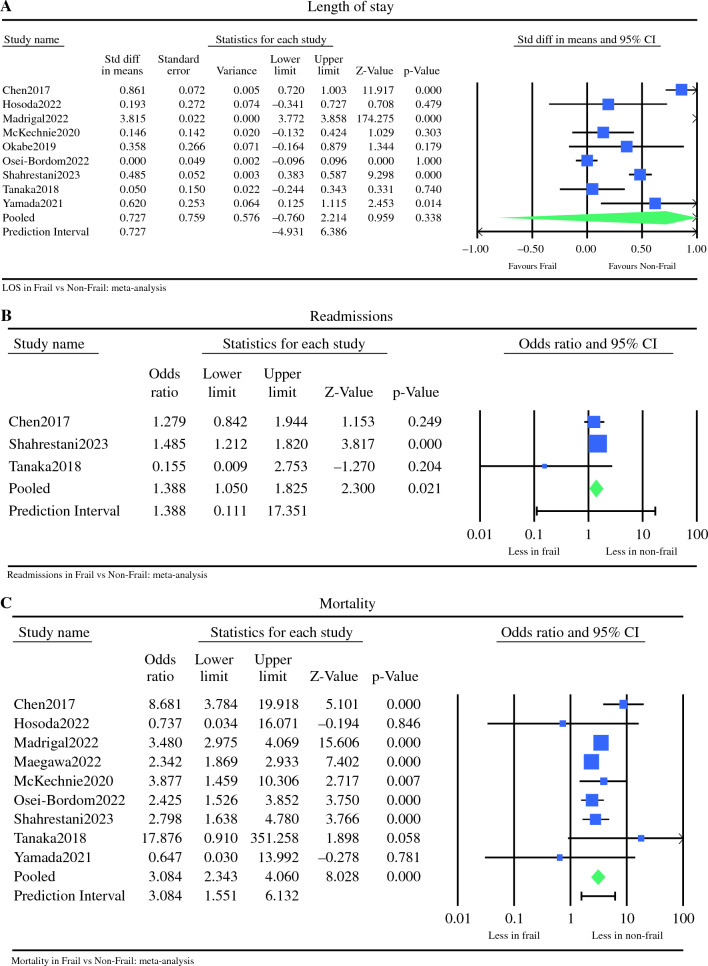
Meta analysis of 30 days outcomes between frail and non-frail patients in terms of (**A**) length of stay, (**B**) readmissions, and (**C**) mortality. Each study is shown by the point estimate of the odds ratio/mean difference (OR; square proportional to the weight of each study) and 95% confidence interval (CI) for the OR (extending lines); the combined ORs/mean difference and 95% CIs by random effects calculations are shown by diamonds. (**A**) F versus NF and length of stay (*n* = 46,949, *p* = 0.338; test for heterogeneity Q = 8898.445, *p* < 0.001, *I*^2^ = 100%). (**B**) F versus NF and readmissions (*n* = 3203, *p* = 0.021; test for heterogeneity Q = 2.697, *p* = 0.260, *I*^2^ = 26%). (**C**) F versus NF and mortality (*n* = 70,956, *p* < 0.001; test for heterogeneity Q = 18.514, *p* = 0.018, *I*^2^ = 57%)

**Fig. 7:**
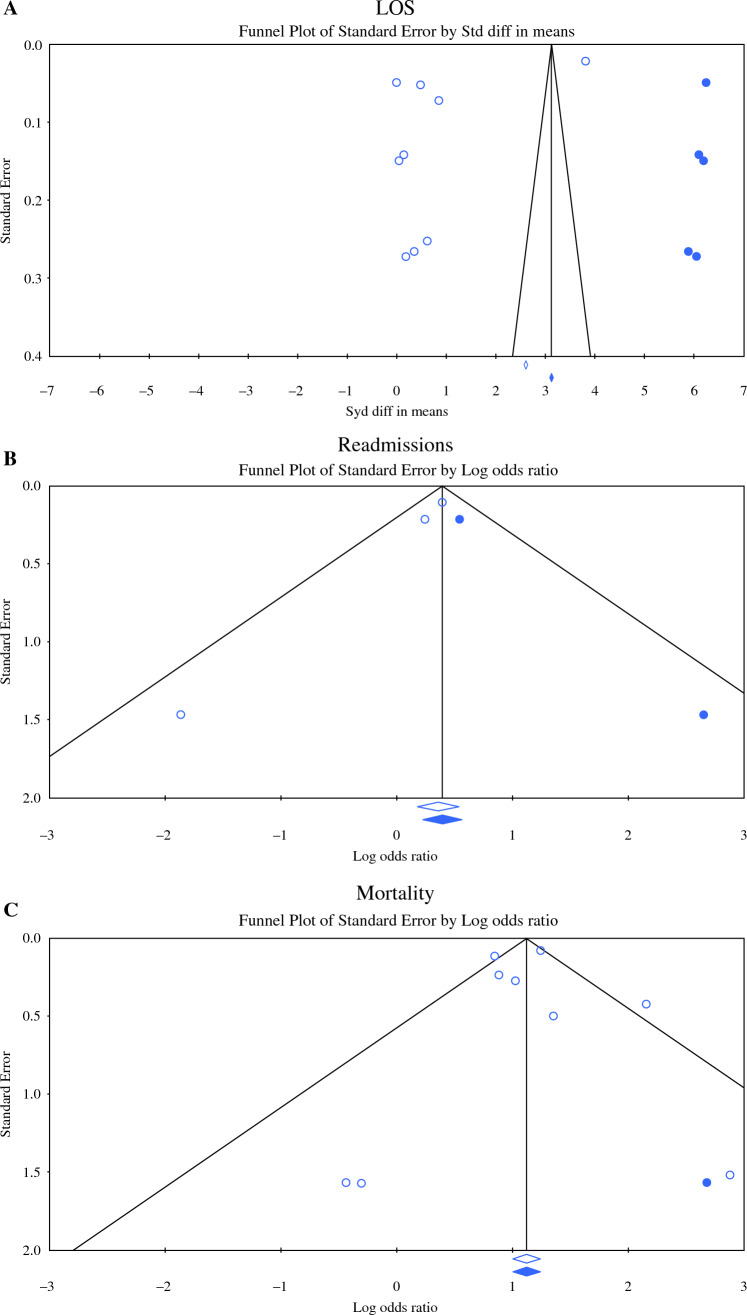
Trim and fill for 30-day outcomes: (**A**) LOS; (**B**) readmissions; (**C**) mortality. The funnel plot is a plot of a measure of study size (usually standard error or precision) on the vertical axis as a function of effect size on the horizontal axis. Large studies appear toward the top of the graph and tend to cluster near the mean effect size. Smaller studies appear toward the bottom of the graph. Through the trim and fill method studies were imputed to adjust for publication bias (full black circles). On the bottom of the graph, the empty diamond shows the OR and confidence interval for the original studies, whereas the full diamond shows the OR and confidence interval for the original and imputed studies

Nine studies, including data on 46,949 patients, provided data on the length of stay.^13–20,22^ There was no significant difference between F and NF patients in terms of postoperative hospital stay, however, with significant interstudy heterogeneity (mean difference 0.727, 95% CI −0.760 to 2.214, *p* = 0.338, Q = 8898.445, *p* < 0.001, *I*^2^ = 100%) (Fig. 6a). Using Duval and Tweedie’s Trim and Fill methods to account for missing studies, five studies were statistically imputed to the right of the mean, reducing funnel plot asymmetry. Using the random effects model, after trim and fill, the imputed point estimate is 2.64350 (95% CI 1.34344–3.94355) (Fig. 7a).

### Readmissions

Three studies, including 3203 patients, provided data on the rate of readmissions between F and NF patients.^13,20,21^ Frail patients had a 1.3 times higher readmissions rate compared with NF patients, without significant interstudy variance (OR 1.388, 95% CI 1.050–1.835, *p* = 0.021, Q = 2.697, *p* = 0.260, *I*^2^ = 26%) (Fig. 6b). Using trim and fill, two studies were imputed to the right of the mean. Using the random effects model the imputed OR is 1.48513 (95% CI 1.14539–1.92564) (Fig. 7b).

### Mortality

Nine studies,^13–17,19–22^ including data on 70,956 patients, compared the mortality rate between F and NF patients. Frail patients had a three times higher 30-day mortality rate compared with NF patients (OR 3.084, 95% CI 2.343–4.060, *p* < 0.001, Q = 18.514, *p* = 0.018, *I*^2^ = 57%) (Fig. 6c). Using trim and fill, one study was imputed to the right of the mean. Using the random effects model the imputed OR is 3.120 (95% CI 2.373–4.102) (Fig. 7c).

## Discussion

Frail patients undergoing oncological liver resections have a higher rate of postoperative morbidity, including surgical, medical complications, and postoperative liver failure, with a longer hospital stay and more frequent readmissions. Frail patients have a significantly higher rate of 30-day mortality compared with nonfrail patients. Comparatively, frail patients are older and show a lower preoperative albumin level, suggestive of a poorer nutritional status.

To our knowledge, this is the first meta-analysis to compare surgical outcomes between frail and nonfrail patients and comes to underscore the importance of in-depth preoperative evaluation of patients undergoing major liver resections. This study provides a statistical synthesis of comparable studies and increases the precision of the overall estimate and the generalizability of the individual results. We agree that the overall results are mostly in agreement with individual studies, but there are conflicting results, such as surgical complications or length of stay where the pooled data provided a balanced overview of the mean effect size. To tackle publication bias, where small studies reporting results in disagreement with large (> 20,000 patients) studies might have not been published, we imputed estimates via trim and fill thus increasing the statistical power of the real-world results. Our meta-analysis, even after trim and fill, agreed with individual studies and provides an objective, unbiased, overview on the role of frailty in predicting outcomes of liver surgery. Frail patients were found to be older and have lower albumin levels; however, this should not be regarded as a potential source of confounding bias, but rather an accompanying characteristic of frail patients; they are usually older and at risk of malnutrition. Frailty does not come to replace age as a criterion to select high-risk patients but to emphasize it and improve prehabilitation, patient and family counselling, operative planning, postoperative care, and will better delineate expectations for clinicians, patients, and their siblings. Although all studies agree frailty is a syndrome characterized by reduced functional reserve and poor response to traumatic stressors, such as surgery, how frailty is defined in each score or tool, variates. While the 11-item modified frailty index (11-mFI) includes cardiac, vascular, or respiratory comorbidities, the Johns Hopkins score focuses more on mobility, strength, weight loss, and exhaustion. Despite having different approaches, all scores have proven similar in identifying and predicting outcomes in an objective, standardized fashion, rather than picking up frail patients by clinical flair—how it is currently done in real practice.^23–27^

Postoperative morbidity after oncological liver resections is high, with a rate up to 30%, and can be even higher when considering subgroups of at-risk patients.^28^ Currently used general scores fail to predict real outcomes in patients. One of the most used in clinical practice, the American College of Surgeons (ACS) National Surgical Quality Improvement Program (NSQIP) score was shown to fail in predicting surgical outcomes after hepatectomy.^29,30^ Similarly, the widely used Portsmouth Physiological and Operative Severity Score for the enumeration of Mortality and morbidity (P-POSSUM) score was unable to adequately predict postoperative outcomes after colorectal cancer liver metastases resection.^31^ A recent systematic review on predictors of outcomes after hepatectomy emphasized the need to establish specialized scores for the subset of patients undergoing liver surgery.^32^ The MELD score can be used to predict liver complications with precision, but we frequently see in our practice patients with major nonliver complications despite having adequate liver function preoperatively. We believe the morbidity and mortality in this scenario can be explained, to an extent, by their frail nature, especially in elderly patients. We need to understand, in numbers, how frailty influences outcomes in order to refine prehabilitation of elderly patients with an otherwise normal liver function. Herein, we show that frailty could be used, if not alone, together with other criteria (e.g., albumin level or nutritional risk scoring) to better predict surgical prognosis.

Quantifying frailty is a hot topic in many specialities, because it can provide a predictable glimpse on the patients biological age with better sensitivity than age alone. With the increasing proportion of older, frail patients requiring surgery for oncological reasons, a set of rules to better stratify the overall operative risks in such groups is welcomed. In our meta-analysis, frailty was associated with a higher rate of overall morbidity and more medical, surgical complications (i.e., bile leaks, wound infection, etc.) and more postoperative liver failure. For this reason, frailty could be used alongside Child Pugh or MELD scores to predict the risk of liver decompensation postoperatively and reduce failure to rescue rates.

From a cost perspective, frailty leads to more frequent readmissions likely related to its associated morbidity. While length of stay did not show a significant difference between the two groups, when adjusting for publication bias, the mean difference shifted to 2.6 days more in the frail group. Still, length of stay can be difficult to appreciate because of interstudy heterogeneity. Finally, postoperative mortality is higher in frail patients, and this should be accounted for when patients and family are counselled preoperatively.

There are limitations to our study. In five studies, frailty was scored retrospectively based on the data available in the prospectively maintained electronic databases.^13–17^ The CFS and the 11-mFI rely on patient reported criteria relating to their independence, mobility, and nutrition, which may have been misinterpreted if not gathered prospectively. This leads confounding and selection bias, which may have influenced the overall results. Also, how frailty is measured varies depending on the score used. This increases heterogeneity in our comparison; however, it is unlikely that one score will be unanimously implemented in future studies if not recommended in international guidelines. For some comparisons, especially for length of stay, data were heterogenous influencing the trim and fill method, which reported publication bias. Despite our efforts to compare the two groups in terms of patients’ characteristics to prove homogeneity between the two groups, not all studies provided equally distributed data on this regard and such the value of it is questionable. Not all patient factors were accounted for in individual studies. Preoperative ASA score, Child Pugh/MELD scores, smoking status, steroid use, or neoadjuvant therapy were not mentioned and are known predictors of morbidity and mortality after liver resections.

## Conclusions

The building body of evidence strongly supports the use of preoperative frailty scoring to stratify patients and aid decision making as it is a strong predictor of morbidity and mortality in patients undergoing oncological liver resections.
